# PDE4D and miR-203 are promising biomarkers for canine atopic dermatitis

**DOI:** 10.1007/s11033-024-09605-3

**Published:** 2024-05-11

**Authors:** Gagandeep Kaur, Chen Xie, Charli Dong, Jonathan Najera, Jeffrey T. Nguyen, Jijun Hao

**Affiliations:** 1 College of Veterinary Medicine, Western University of Health Sciences, Pomona, CA USA; 2 Animal Dermatology Clinic, Pasadena, CA USA

**Keywords:** Canine, Atopic dermatitis, Biomarkers, MiR-203, Cytokine, Pde4d

## Abstract

**Background:**

Canine atopic dermatitis (CAD) is a common genetically predisposed, inflammatory, and pruritic skin disorder that affects dogs globally. To date, there are no specific biomarkers available to diagnose CAD, and the current diagnosis is based on a combination of criteria including patient history, clinical signs, and exclusion of other relevant differential diagnoses.

**Methods and results:**

We examined the gene expression of phosphodiesterase 4D (PDE4D) in peripheral blood mononuclear cells (PBMCs), as well as miR-203 and miR-483 in plasma, in three groups: healthy dogs, CAD dogs, and other inflammatory pruritic skin diseases (OIPSD) such as pemphigus foliaceus, scabies, cutaneous lymphoma, and dermatophytosis. Our results showed that PDE4D gene expression in the CAD group is statistically higher compared to those in the healthy and OIPSD groups, suggesting PDE4D may be a specific marker for CAD. Nevertheless, no correlation was found between PDE4D gene expression levels and the lesion severity gauged by CAD severity index-4 (CADESI-4). We also showed that miR-203 is a generic marker for clinical dermatitis and differentiates both CAD and OIPSD inflammatory conditions from healthy controls.

**Conclusions:**

We show that PDE4D is a potential marker to differentiate CAD from non-atopic healthy and OIPSD while miR-203 may be a potential marker for general dermatologic inflammation. Future study of PDE4D and miR-203 on a larger scale is warranted.

**Supplementary Information:**

The online version contains supplementary material available at 10.1007/s11033-024-09605-3.

## Introduction

CAD is a common genetically predisposed, inflammatory and pruritic allergic skin disorder involving cutaneous inflammation, skin barrier dysfunction, secondary infections, and hypersensitivity to environmental allergens [1]. Diagnosis of CAD is based on a combination of criteria such as patient history, clinical signs, and exclusion of other relevant differential diagnosis [2]. CAD affects 3–15% of the canine population worldwide with higher risk of developing the disease in urban settings [3]. To date, there are no specific biomarkers available for CAD. Development of reliable, less invasive, and efficient biomarkers for swift diagnosis of CAD is highly sought after. Studies in humans indicate that interleukin-31 may be a diagnostic marker for chronic pruritus [4]. Administration of IL-31 induces pruritic behaviors in dogs, and its levels are elevated in the majority of dogs with CAD [5]. Recently, Asahina et al. reported that serum thymus and activation-regulated chemokine (TARC) concentrations were significantly higher in dogs with CAD than in healthy dogs, and its concentrations decreased in treated dogs with the attenuation of clinical signs [6]. Considering the complex pathophysiology and heterogeneity of CAD, it is possible that multiple biomarkers exist for CAD [7]. However, all the published studies focus on comparison of CAD to healthy controls, and there are no studies of specific biomarkers available in literature to distinguish CAD from other pruritic dermatologic diseases such as, dermatophytosis, ectoparasites, pemphigus foliaceus, and cutaneous lymphoma.

In our previous study, we have shown that gene expression of PDE4D in PBMCs and levels of miR-203 and miR-483 in plasma are significantly elevated in CAD dogs when compared to healthy counterparts [8]. The PDE4D is one of the isoforms of phosphodiesterase 4 and is involved in a variety of epithelial functions including skin barrier protection [9]. MicroRNAs (miRNAs) interfere with mRNA translation and can be important biomarkers for various inflammatory and autoimmune disease conditions [10]. Our previous results on PDE4D, miR-203 and miR-483 are promising; however, further studies to examine these potential markers comparing CAD to other similar inflammatory dermatologic diseases in dogs is needed. We therefore hypothesize that PDE4D, miR-203 and / or miR-483 could be potential specific biomarker(s) for CAD. In this study, we investigated if expression levels of PDE4D in PBMCs and miR-203 and miR-483 in plasma are specific and sensitive to CAD, as well as, if expression levels correlated with clinical sign severity by parameter comparisons among dogs with CAD, dogs with OIPSD and healthy dogs.

## Materials and methods

### Inclusion criteria for healthy dogs

 Dogs greater than one year of age, a body condition score of at least 4 on a 9-point scale, with no history or clinical signs of pruritus or immune modulating disease conditions were enrolled in the study. These dogs were examined and had normal physical examination findings, as well as complete blood counts and blood chemistries within the last twelve months. Only dogs that were current on ectoparasite control, did not have the history or clinical signs of pruritus, did not receive any medications for conditions related to pruritus, were not treated for any immune related conditions, and did not receive any immune modulating drugs over the course of last twelve months were included in this group.

### Inclusion criteria for CAD dogs

Dogs greater than one year of age and a body condition score of at least 4 on a 9-point scale were enrolled in this category. Clinical diagnosis of CAD in enrolled dogs was based on detailed patient history, clinical signs, and exclusion of other possible skin pathologies that can present as CAD. History included the age of onset, non-lesional prior to clinical signs, seasonal component, and/or a veterinary prescribed elimination diet trial was performed prior to enrollment. Clinical signs for inclusion were as outlined in Hensel et al. [11], : pruritus, erythema, papules, or self-trauma to the face, concave aspect of pinnae, ventrum, axillae, inguinal region, perineal region, and distal extremities. Within the CAD group, animals were categorized as mild (< 10), moderate (11–35), or severe (> 60) based on the CADESI-4 values [12]. Flea combing, skin scrapings, and skin cytologies were performed. Underlying systemic diseases were ruled out through physical examinations and serum chemistry and hematology analyses.

### Exclusion criteria for CAD dogs

Clinical evidence of ectoparasite infestations (flea allergy dermatitis, scabies etc.), concurrent bacterial or fungal cutaneous infections, and sole food allergies, resulted in exclusion from the study. Patients must not have undergone immunotherapy prior or during enrollment. In addition, patients could not have taken steroids, cyclosporine, oclacitinib, lokivetmab, antihistamines, antibiotics, and antifungals within one month before enrollment.

### Inclusion criteria for OIPSD

Dogs older than one year of age and diagnosed with other inflammatory diseases that could present similarly CAD, specifically pemphigus foliaceus, scabies, cutaneous lymphoma and dermatophytosis were enrolled in this group. Patients that presented with pruritus and fulfilled practical guidelines for definitive diagnoses of these skin conditions as listed below were enrolled. For scabies, clinical signs of pruritus and demonstration of mites in skin scrapings were used to confirm the diagnosis [13]. For cutaneous lymphoma, the diagnosis was confirmed based on the histopathological skin biopsy samples [14]. For dermatophytosis, presence of an active infection was confirmed by Wood’s lamp and direct examination to confirm active hair infection, positive dermatophyte culture and/or ringworm (Dermatophyte) by RT-PCR [15]. Diagnoses for pemphigus complex was confirmed by clinical signs and histopathological examinations [16]. Routine hematology and serum chemistry analysis, total T4, dermatologic database, such as skin cytology, skin scrapings and fungal cultures were performed for all the patients in this group to rule out co-morbidities.

### Exclusion criteria for OIPSD

Other ectoparasite infestations such as fleas, flea allergy dermatitis, primary bacterial or fungal cutaneous infections, exclusive food allergies, or systemic diseases resulted in exclusion from the study. Patients could not have taken steroids, cyclosporine, oclacitinib, lokivetmab, antihistamines antibiotics, and antifungals within one month before enrollment.

### PBMC cells isolation

PBMCs were isolated from 4mL of whole blood collected in EDTA vacutainers and diluted with Phosphate Buffered Saline (PBS) at a 1:3 ratio. The diluted whole blood was layered on top of Ficoll-Paque PLUS (GE Healthcare) and centrifuged at 2500 rpm for 25 min with no brake. The PBMCs interphase was transferred to a 1.5 ml centrifuge tube followed by centrifuging at 1500 rpm for five minutes. The PBMCs pellet was collected and stored at -80 ºC for future use.

### RNA extraction and real-time PCR

RNA was extracted from PBMCs by using the RNeasy Mini Kit (Qiagen) following the manufacturer’s protocol with an additional DNAse I digestion step. The first strand cDNA was synthesized using the High-Capacity cDNA Reverse Transcription Kit (Applied Biosystems) according to the manufacturer’s instructions. The real-time-PCR reactions were performed using Fast Syber Green Master Mix (Applied Biosystems) in triplicate on Bio-Rad CFX connected Real-Time PCR system. Canine glyceraldehyde-3-phosphate dehydrogenase (GAPDH) gene was used as an internal control. The primer sets designed for real-time PCR are: Canine PDE4D 5’-AATCACAGGTGGGCTTCATAG-3’, 5’-CACTGCAGCTAGTGTCTTCTT-3’; Canine GAPDH5`-GGAGAAAGCTGCCAAATATG-3’, 5’-ACCAGGAAATGAGCTTGACA-3’.

### MicroRNA extraction and real-time PCR

The 2 mL of whole blood collected with EDTA coated tubes was centrifuged at 2500 rpm for 10 min and the plasma supernatant containing miRNA was collected. miRNeasy Serum/Plasma Kit (Qiagen) was used to extract microRNA, and the miRNeasy Serum/Plasma Spike-In Control (Qiagen) was added during the process. The reverse transcription was conducted by following the protocol of “TaqMan Small RNA Assays” (Applied Bio-systems), which were performed in triplicate on Bio-Rad CFX connected Real-Time PCR system by using TaqMan Universal PCR Master Mix (Applied Biosystems) according to the manufacturer’s instructions. The data were normalized to the internal control miR-39. The following TaqMan probe and primer sets (ThermoFisher) were used: miR-39 (RT 000200), miR-203 (RT 000507), and miR-483 (RT 002560).

### Statistical analysis

All values were expressed as means ± SE. Comparison of the means between two groups was conducted using Student’s t-test with GraphPad Prism 10.2.0 (GraphPad, San Diego, CA, USA), and the results of multiple groups were compared using a one-way analysis of variance (ANOVA). The results were considered statistically significant if the p-value was less than 0.05.

## Results and discussion

### PDE4D gene expression is upregulated in CAD dogs compared to the heathy and OIPSD dogs

**Fig. 1:**
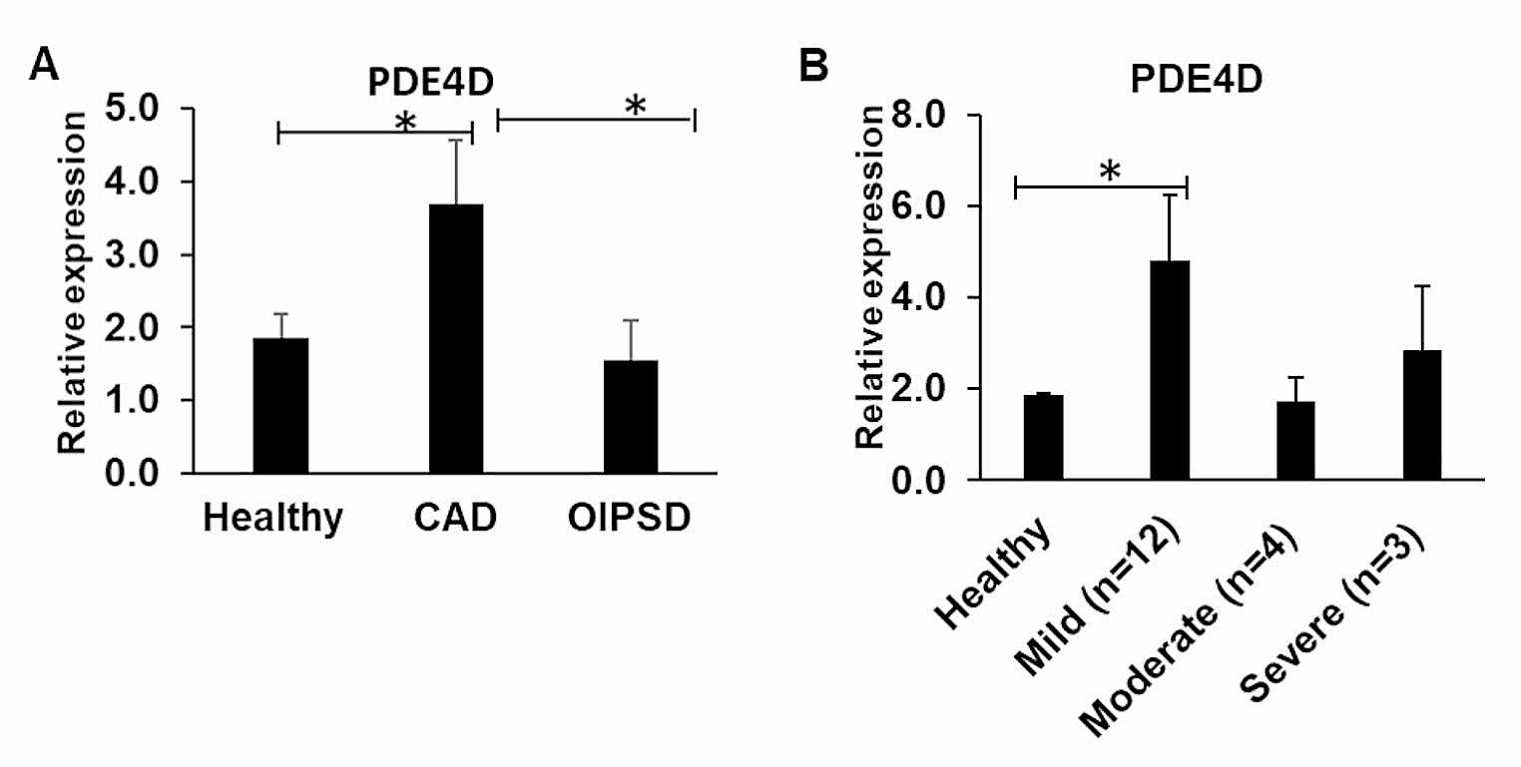
PDE4D gene expression of PBMCs in the heathy dogs, CAD dogs and OIPSD dogs.(**A**) RT-PCR result indicates that PDE4D gene expression is statistically significantly upregulated statistically significantly in CAD dogs compared to the Heathy and OIPSD dogs from healthy (*p < 0.05). (**B**) RT-PCR result shows that PDE4D gene expression in the mild CAD category, but not the moderate or severe CAD categories, is statistically significantly increased compared to the healthy group

We enrolled 19 healthy dogs, 19 CAD dogs, and 5 OIPSD dogs based on the outlined inclusion and exclusion criteria (Supplementary Table S1). Levels of PDE4D gene expression in PBMCs were compared between the healthy, CAD, and OIPSD groups. The PDE4D expression in the CAD group was statistically increased by 1.98-fold and 2.38-fold in comparison to the healthy group and the OIPSD group, respectively (Fig. 1A, p-value < 0.05). We further analyzed the PDE4D gene expression levels among healthy, and mild CAD (*n* = 12; CADESI-4 < 35), moderate CAD (*n* = 4; CADESI-4 35–59) and severe CAD (*n* = 3; CADESI-4 > 60) groups (Fig. 1B). In comparison to the healthy group, PDE4D gene expression in the mild CAD group was statistically significantly increased by 2.57-fold. However, there was no dramatic difference of PDE4D gene expression in either the moderate or severe CAD categories when compared to healthy dogs. This data indicates that PDE4D could differentiate between CAD and healthy or OIPSD conditions, but it could not distinguish the severity levels of CAD.

PDE4D is one isoform of PDE4, a predominant enzyme that metabolizes cyclic adenosine monophosphate and regulates pro- and anti-inflammatory activities of various immune cells [9]. PDE4 plays a pivotal role in epithelial functions including skin barrier protection [19]. More recent studies have shown that PDE4 is implicated in AD pathophysiology and inhibition of PDE4 is beneficial to both human AD and CAD [20–24]. Our results reveal an elevated expression of PDE4D in AD dogs compared to healthy controls and OIPSD patients, suggesting its potential as a biomarker to differentiate AD from other similar inflammatory dermatological conditions. Nevertheless, our data indicates a statistically significant increase in PDE4D gene expression only in mild CAD dogs, not the moderate and severe CAD dogs. This could be attributed to two possibilities. Firstly, PDE4D might specifically serve as a biomarker for mild CAD, consistent with the effectiveness of the PDE4 inhibitor crisaborole drug in treating mild and moderate AD in human patients. Secondly, our stringent criteria for patient enrollment, excluding those treated with steroids, cyclosporine, oclacitinib, lokivetmab, antihistamines, antibiotics, and antifungals within one month, resulted in smaller sample sizes for moderate (*n* = 4) and severe (*n* = 3) CAD groups compared to the mild CAD (*n* = 12) group.

### Plasma MiR-203 is statistically significantly increased in the AD group in comparison to the healthy group, but not the OIPSD group

**Fig. 2:**
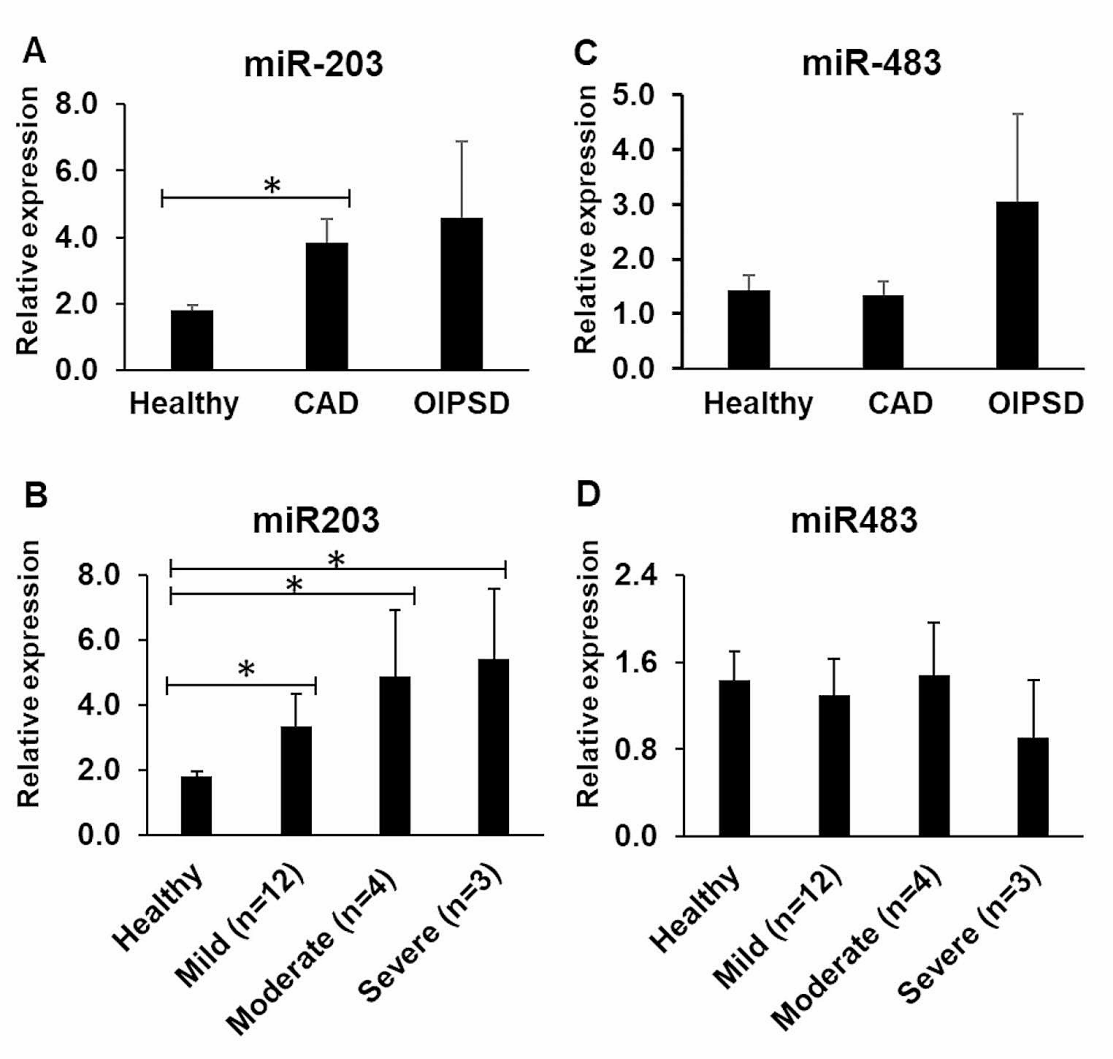
Expression of plasma MiR-203 and miR483 in the heathy dogs, CAD dogs and OIPSD dogs. (**A**) MiR-203 is statistically significantly increased in the CAD group in comparison to the healthy group, but not the OIPSD group. (**B**) RT-PCR indicates that miR-203 levels are statistically significantly higher in the moderate and severe categories of the CAD groups compared to healthy group. (**C**) RT-PCR result does not show any statistically significant changes for miR483 in both CAD and OIPSD patient groups compared to the healthy group. (**D**) There is no significant difference for miR483 between the healthy group and either the mild, moderate or severe categories in the CAD groups

Plasma levels of miR-203 were compared between the healthy, CAD, and OIPSD groups. The miR203 level in the CAD group was increased by 2.1-fold in comparison to the healthy group (Fig. 2A, p-value < 0.05). But there was no statistically significant difference in the miR-203 levels between the CAD and OIPSD groups. For the mild, moderate, and severe categories within the CAD group, miR-203 levels were significantly higher in the mild (1.8-fold), moderate (2.7-fold) and severe (3.0-fold) CAD groups compared to healthy dogs (Fig. 2B, p-values < 0.05). This data suggests that miR-203 could distinguish CAD and OIPSD patients from healthy but not CAD patients from OIPSD patients, and hence it may be a general marker for skin inflammation. In addition, there were no statistically significant changes for miR483 levels in both CAD and OIPSD patient groups compared to the healthy control group, or amongst the mild, moderate and severe CAD groups, excluding the biomarker potential of plasma miR483 in CAD diagnosis (Fig. 2C and 2D).

MiRNAs interfere with mRNA translation and are important biomarkers for various disease conditions [10]. Notably, miR-203 is upregulated in the serum of children with AD, and we have previously shown the increased levels of miR-203 in the plasma of CAD dogs compared to healthy controls, suggesting its potential as a CAD biomarker [8]. Our current study confirmed the same expression trend of miR-203. However, no significant difference in miR-203 levels between CAD and OIPSD groups was identified in this study, suggesting that miR-203 may serve as a generic marker for skin-related inflammation rather than a specific marker for CAD. The increased expression of miR-203 in the plasma of CAD dogs may be associated with down-regulation of the miR-203 target SOCS-3 [25].

The limited patient sample sizes, particularly in the OIPSD group, were attributed to several factors including the widespread use of medications for external parasites, strict inclusion criteria, and medication withdrawal period. These factors led to the exclusion of many potential participants, resulting in a lower OIPSD enrollment number. The reduced sample size of the OIPSD group could impact the statistical reliability of biomarkers, and a further study of those potential biomarkers on a large scale is warranted.

## Conclusion

To date, there are no specific biomarkers available to diagnose CAD, and the current guideline-based diagnosis of CAD is non-specific and time consuming, hindering early intervention for the disease or even leading to inaccurate diagnoses. Here, we show that PDE4D is a potential marker to differentiate CAD from non-atopic healthy and OIPSD while miR-203 may be a potential marker for general dermatologic inflammation. Future study of PDE4D and miR-203 on a larger scale is warranted.

## Electronic supplementary material

Below is the link to the electronic supplementary material.

Supplementary Material 1

## Data Availability

Data is provided within the manuscript or supplementary information files.
