# Supplementary material for: PDE4D and miR-203 are promising biomarkers for canine atopic dermatitis
Source: Mol Biol Rep. 2024 May 11;51(1):651. doi: 10.1007/s11033-024-09605-3 (PMC11088561; doi:10.1007/s11033-024-09605-3)
Supplement: Supplementary file 1 — Supplementary Material 1 [file 11033_2024_9605_MOESM1_ESM.docx]

**Supplementary Table 1. Age at enrollment, sex, breed and CADESI-4 for dogs enrolled in the study.**

| **CAD patients** | **Age (year)** | **Sex** | **Breed** | **CADESI-4** |
| --- | --- | --- | --- | --- |
| Mild | 7 | MN | Terrier Mix | 10 |
|  | 1 | FS | Shih Tzu | 10 |
|  | 1 | M | Pug | 11 |
|  | 5 | FS | Welsh Corgi | 12 |
|  | 1 | FS | Corgi | 12 |
|  | 1 | MN | German Shepherd Dog Mix | 13 |
|  | 8 | F | German Shepherd Dog Mix | 14 |
|  | 5 | FS | Mixed Breed | 17 |
|  | 7 | FS | Labradoodle | 20 |
|  | 1 | FS | Australian Cattle Dog | 26 |
|  | 3 | M | French Bulldog | 26 |
|  | 12 | MN | West Highland White Terrier | 30 |
| Moderate | 1 | MN | Staffordshire Bull Terrier | 38 |
|  | 2 | MN | French Bulldog | 43 |
|  | 2 | MN | Australia Shepherd | 48 |
|  | 2 | FS | Golden Retriever | 56 |
| Severe | 8 | FS | Mixed Breed | 62 |
|  | 3 | F | Husky-Lab mix | 72 |
|  | 7 | FS | Chihuahua | 93 |

| **OIPSD patients** | **Age (year)** | **Sex** | **Breed** | **CADESI-4** | **Disease** |
| --- | --- | --- | --- | --- | --- |
|  | 7 | MN | Golden Retriever | 66 | Cutaneous Lymphoma |
|  | 4 | MN | Manchester Terrier | 27 | Dermatophyte |
|  | 5 | N | Maltipoo | 101 | Scabies |
|  | 2 | MN | Pomeranian Mix | 2 | Pemphigus Foliaceus |
|  | 11 | FS | Pit Bull Mix | 64 | Pemphigus Foliaceus |

| **Healthy**  **Control** | **Age (year)** | **Sex** | **Breed** |
| --- | --- | --- | --- |
|  | 5 | FS | Mixed Breed |
|  | 4 | FS | German Shepherd |
|  | 8 | MN | Boxer Mix |
|  | 6 | FS | Boxer |
|  | 12 | FS | Golden Retriever |
|  | 8 | MN | Yorkshire Terrier |
|  | 3 | MN | Miniature Poodle |
|  | 1 | MN | Boxer |
|  | 1 | FS | Boxer |
|  | 8 | MN | Siberian Husky |
|  | 3 | FS | Sheepadoodle |
|  | 10 | FS | Beagle |
|  | 7 | MN | Bassett Hound |
|  | 6 | FS | Terrier Mix |
|  | 6 | M | Shih Tzu |
|  | 3 | FS | Pitbull |
|  | 8 | FS | Labrador Mix |
|  | 10 | MN | Australian Shepherd |
|  | 2 | MN | Shepherd Mix |
